# Supplementary material for: Analysis of Conservative Treatment Trends for Lumbar Disc Herniation with Radiculopathy in Korea: A Population-Based Cross-Sectional Study
Source: Healthcare (Basel). 2023 Aug 21;11(16):2353. doi: 10.3390/healthcare11162353 (PMC10454101; doi:10.3390/healthcare11162353)
Supplement: Supplementary file 1 [file healthcare-11-02353-s001.zip › healthcare-2536443-supplementary.pdf]

Supplementary Material

Supplementary Table S1. Classification of medication.

Category	Anatomical Therapeutic Chemical Classification Code
Opioids	N02A (opioids)
Non-opioid analgesics	M01A (anti-inflammatory and antirheumatic products, non-steroids), M01C (specific antirheumatic agents), M02A (topical products for joint and muscular pain), M03 (muscle relaxants),<br>N02B (other analgesics and antipyretics), N03A (anti-epileptics)
Aesthetics	N01 (anesthetics)
Gastrointestinal	A02AA (magnesium compounds), A02AA02 (magnesium oxide), A02AA04 (magnesium hydroxide), A02AB (aluminum compounds), A02AB01 (aluminum hydroxide), A02AB03 (aluminum phosphate), A02AC01 (calcium carbonate), A02AD01 (ordinary salt combinations), A02AD03 (almagate), A02AD04 (hydrotalcite), A02AD05 (almasilate), A02AX (antacids, other combinations),<br>A02B (drugs for peptic ulcer and gastro-esophageal reflux disease (GORD)), A02X (other drugs for acid-related disorders), A03 (drugs for functional gastrointestinal disorders),<br>A06 (drugs for constipation), A07 (antidiarrheals, intestinal anti-inflammatory/anti-infective agents), A09 (digestives, incl. enzymes), A16 (other alimentary tract and metabolism products)
Antipsychotics	N05 (psycholeptics), N06 (psychoanaleptics), N07 (other nervous system drugs)
Antibiotics	D01 (antifungals for dermatological use), D06A (antibiotics for topical use), D06BB (sulfonamides and other antibacterials for topical use), D06BX (other topical antibiotics),<br>G01A (anti-infectives and antiseptics for gynecological use),<br>J01 (antibacterials for systemic use), J02 (antimycotics for systemic use), J04 (drugs for treatment of tuberculosis), J05 (antivirals for systemic use)
Steroids	D07 (corticosteroids for topical use), H02 (corticosteroids for systemic use)
Others	A01A (stomatological preparations), A05 (bile and liver therapy), A10 (drugs used in diabetes), A11 (vitamins), A12 (mineral supplements),<br>B01A (antithrombotic agents), B02 (antihemorrhagics), B03 (anti-anemic preparations), B05 (blood substitutes and perfusion solutions),<br>C (cardiovascular system),<br>D02A (emollients and protectives), D03A (preparations for treatment of wounds and ulcers), D05 (antipsoriatics), D06 (antibiotics and chemotherapeutics for dermatological use), D08 (antiseptics and disinfectants), D11 (other dermatological preparations),<br>G02 (other gynecologicals), G03 (sex hormones and modulators of the genital system), G04 (urologicals),<br>H01 (pituitary and hypothalamic hormones and analogues), H03 (thyroid therapy), H05 (calcium homeostasis),<br>J06 (immune sera and immunoglobulins),<br>L (antineoplastic and immunomodulating agents), M04 (antigout preparations), M05 (drugs for treatment of bone diseases), M09 (other drugs for disorders of the musculo-skeletal system), N02 (analgesics), N04 (anti-Parkinson's drugs), P (antiparasitic products, insecticides, and repellents), R (respiratory system), S (sensory organs), V (various)

Supplementary Table S2. Annual average KRW–USD exchange rate and healthcare price index.

Year	KRW/USD	Healthcare and medical service price index
2010	1156	0.9164
2011	1107.99	0.9325
2012	1126.76	0.9410
2013	1095.04	0.9444
2014	1053.12	0.9510
2015	1131.52	0.9629
2016	1160.41	0.9725
2017	1130.48	0.9810
2018	1100.58	0.9805
2019	1166.11	0.9851
2020	1180.01	1.0000

Notes: this information is available on the Korean Statistical Information Service website (http://kosis.kr), which represents the relative price levels of adjusted costs, as of 2020.

Supplementary Table S3. Medical use and cost of non-surgical treatment for lumbar radiculopathy.

Year	Category	Korean medicines	Medication	Nerve blocks	Others	Physiotherapy	TPI
2010	No. of patients (%)	1,185 (5.95%)	16,149 (81.12%)	4,636 (23.29%)	8,952 (44.97%)	12,103 (60.80%)	503 (2.53%)
	Medical costs	USD 73,924	USD 344,307	USD 403,233	USD 53,246	USD 592,613	USD 9,720
2011	No. of patients (%)	1,379 (6.50%)	17,173 (80.96%)	4,991 (23.53%)	9,018 (42.51%)	12,772 (60.21%)	465 (2.19%)
	Medical costs	USD 116,598	USD 378,949	USD 504,719	USD 62,386	USD 634,556	USD 7,555
2012	No. of patients (%)	1,393 (6.19%)	18,252 (81.04%)	6,023 (26.74%)	8,961 (39.79%)	12,918 (57.36%)	357 (1.59%)
	Medical costs	USD 123,233	USD 360,584	USD 679,677	USD 56,367	USD 604,935	USD 6,505
2013	No. of patients (%)	1,480 (6.45%)	18,477 (80.49%)	6,265 (27.29%)	9,032 (39.34%)	12,974 (56.51%)	412 (1.79%)
	Medical costs	USD 138,954	USD 350,197	USD 757,890	USD 64,000	USD 644,952	USD 7,187
2014	No. of patients (%)	1,522 (6.48%)	18,916 (80.51%)	6,978 (29.70%)	8,759 (37.28%)	12,993 (55.30%)	393 (1.67%)
	Medical costs	USD 156,910	USD 390,679	USD 957,798	USD 74,842	USD 686,472	USD 6,555
2015	No. of patients (%)	1,495 (6.24%)	19,319 (80.69%)	7,377 (30.81%)	8,506 (35.53%)	13,049 (54.50%)	408 (1.70%)
	Medical costs	USD 153,504	USD 373,665	USD 1,013,400	USD 72,617	USD 614,573	USD 7,705
2016	No. of patients (%)	1,388 (5.69%)	19,807 (81.17%)	8,058 (33.02%)	8,303 (34.03%)	12,993 (53.25%)	386 (1.58%)
	Medical costs	USD 142,713	USD 419,074	USD 1,063,903	USD 70,252	USD 606,109	USD 6,074
2017	No. of patients (%)	1,329 (5.39%)	20,114 (81.52%)	8,541 (34.61%)	8,256 (33.46%)	12,859 (52.11%)	397 (1.61%)
	Medical costs	USD 149,261	USD 432,011	USD 1,218,973	USD 78,968	USD 623,613	USD 6,205
2018	No. of patients (%)	1,420 (5.61%)	20,682 (81.72%)	8,810 (34.81%)	8,184 (32.34%)	12,642 (49.95%)	418 (1.65%)
	Medical costs	USD 156,590	USD 504,103	USD 1,391,060	USD 95,328	USD 631,604	USD 8,805
2019	No. of patients (%)	1,801 (6.81%)	21,432 (81.05%)	9,349 (35.36%)	7,695 (29.10%)	12,549 (47.46%)	416 (1.57%)
	Medical costs	USD 292,438	USD 566,898	USD 1,500,957	USD 87,162	USD 609,558	USD 8,196

TPI: trigger point injection.

Supplementary Table S4. Medical use of different nerve blocks.

Year	Category	Epidural nerve block	Peripheral branch of the spinal nerve block	Spinal nerve plexus, root, or ganglion block
2010	No. of patients (%)	2,727 (14.06)	1,465 (7.55)	1,239 (6.39)
	Medical costs	USD 179,812	USD 86,832	USD 132,759
2011	No. of patients (%)	2,917 (14.10)	1,496 (7.23)	1,408 (6.81)
	Medical costs	USD 226,420	USD 81,314	USD 192,010
2012	No. of patients (%)	3,351 (15.23)	1,606 (7.30)	2,024 (9.20)
	Medical costs	USD 263,107	USD 92,430	USD 316,911
2013	No. of patients (%)	3,410 (15.24)	1,491 (6.67)	2,345 (10.48)
	Medical costs	USD 259,610	USD 88,157	USD 404,073
2014	No. of patients (%)	3,432 (15.02)	1,599 (7.00)	2,985 (13.07)
	Medical costs	USD 286,329	USD 106,050	USD 555,241
2015	No. of patients (%)	3,446 (14.78)	1,602 (6.87)	3,500 (15.01)
	Medical costs	USD 257,159	USD 95,205	USD 650,126
2016	No. of patients (%)	3,590 (15.12)	1,647 (6.94)	4,010 (16.89)
	Medical costs	USD 269,464	USD 101,281	USD 681,839
2017	No. of patients (%)	3,769 (15.65)	1,646 (6.84)	4,397 (18.26)
	Medical costs	USD 279,889	USD 109,579	USD 816,236
2018	No. of patients (%)	3,682 (14.96)	1,715 (6.97)	4,828 (19.62)
	Medical costs	USD 292,612	USD 120,707	USD 961,053
2019	No. of patients (%)	3,558 (13.84)	1,847 (7.18)	5,442 (21.17)
	Medical costs	USD 277,771	USD 130,504	USD 1,073,605
